# Dietary Intake of Curcumin Improves eIF2 Signaling and Reduces Lipid Levels in the White Adipose Tissue of Obese Mice

**DOI:** 10.1038/s41598-018-27105-w

**Published:** 2018-06-13

**Authors:** Masuko Kobori, Yumiko Takahashi, Hiroaki Takeda, Masatomo Takahashi, Yoshihiro Izumi, Yukari Akimoto, Mutsumi Sakurai, Hideaki Oike, Toshiyuki Nakagawa, Masanori Itoh, Takeshi Bamba, Toshiyuki Kimura

**Affiliations:** 1 Food Research Institute, National Agriculture and Food Research Organization, Tsukuba, Ibaraki 305-8642 Japan; 2 Medical Institute of Bioregulation, Kyushu University, Fukuoka, Fukuoka, 812-8582 Japan; 3 Department of Neurobiology, Gifu University Graduate School of Medicine, Gifu, Gifu, 501-1194 Japan

## Abstract

White adipose tissue (eWAT) plays a crucial role in preventing metabolic syndrome. We aimed to investigate WAT distribution and gene expression and lipidomic profiles in epididymal WAT (eWAT) in diet-induced obese mice, reflecting a Western-style diet of humans to elucidate the bioactive properties of the dietary antioxidant curcumin in preventing lifestyle-related diseases. For 16 weeks, we fed C57BL/6J mice with a control diet, a high-fat, high-sucrose and high-cholesterol Western diet or Western diet supplemented with 0.1% (*w*/*w*) curcumin. Although the dietary intake of curcumin did not affect eWAT weight or plasma lipid levels, it reduced lipid peroxidation markers’ levels in eWAT. Curcumin accumulated in eWAT and changed gene expressions related to eukaryotic translation initiation factor 2 (eIF2) signalling. Curcumin suppressed eIF2α phosphorylation, which is induced by endoplasmic reticulum (ER) stress, macrophage accumulation and nuclear factor-κB (NF-κB) p65 and leptin expression, whereas it’s anti-inflammatory effect was inadequate to decrease TNF-α and IFN-γ levels. Lipidomic and gene expression analysis revealed that curcumin decreased some diacylglycerols (DAGs) and DAG-derived glycerophospholipids levels by suppressing the glycerol-3-phosphate acyltransferase 1 and adipose triglyceride lipase expression, which are associated with lipogenesis and lipolysis, respectively. Presumably, these intertwined effects contribute to metabolic syndrome prevention by dietary modification.

## Introduction

Curcumin is a major yellow pigment of the spice turmeric, and it possesses antioxidant and anti-inflammatory activities, which has been intensely investigated for therapeutic use in cancer, Alzheimer’s disease, and other chronic diseases^1–4^. However, Nelson *et al*. recently reported that curcumin was classified as a pan-assay interference compound and an invalid metabolic panacea candidate while screening lead compounds for drug development; besides, they described curcumin as an unstable, reactive, non-bioavailable compound, and no double-blinded, placebo-controlled clinical trial of the agent has been successful to date^3^. Conversely, such low specific manifold bioactivity and low bioavailability are established properties of antioxidant polyphenolic food components^5^. Some prospective studies revealed that some classes of polyphenols prevent lifestyle-related diseases, perhaps, through anti-oxidative and anti-inflammatory properties^6–9^. One such polyphenol is flavonoid quercetin, the median estimated intake of which is 15.5 mg/day in Japan compared with the estimated mean curcumin intake of 2.7–14.7 mg/day in Korea^10,11^. As the dietary intake of curcumin is analogous to flavonoid, curcumin might prevent lifestyle-related diseases through dietary life.

The habitual intake of a Western-style diet, characterised by high consumption of red and processed meat, high-fat dairy products and sweets, is an established factor in developing obesity and metabolic syndrome^12^. Previously, we reported that the dietary intake of anti-oxidative flavonoid quercetin exacerbated obesity and metabolic syndrome in Western diet-fed mice^13,14^. Apparently, a comprehensive analysis is essential to elucidate various physiological functions of food components. Thus, using a comprehensive gene expression analysis, we demonstrated that the dietary intake of quercetin transformed the gene expression profile of epididymal white adipose tissue (eWAT) in Western diet-induced obese mice^13,14^. WAT, crucial for triglyceride storage and the regulation of glucose homeostasis, plays a vital role in the development of obesity-induced insulin resistance and metabolic syndrome. Excessive triglyceride accretion and hypertrophy in WAT resulted in hypoxia and oxidative stress and induced chronic low-grade inflammation by triggering the production of adipocyte-derived mediators, classical activation of M1 macrophages and recruitment of immune cells that produce type 1 responses^15^. Reportedly, type 1 cytokines, such as TNF-α and IFN-γ, produced by adipocytes, M1 macrophages and other immune cells promote insulin resistance in WAT and other tissues^15^.

Besides, glucronised and sulphated curcumin were detected in the plasma of human subjects after the oral administration of curcumin. Vareed *et al*. reported that the maximum blood concentration of curcumin conjugates and the half-lives were 0.71–7.04 μg/mL and 3.19–14.46 h after the administration of 10-g curcumin in healthy subjects, respectively^16^. Nevertheless, the bioavailability was minimal, and curcumin, as well as quercetin, reportedly possessed a potent scavenging ability of the reactive oxygen species and suppressed the inflammation through oxidative stress-inducible signalling, such as nuclear factor-κB (NF-κB), Janus kinase (JAK)/signal transducers and activators of transcription and mitogen-activated protein kinase (MAPK) signalling in some cell and animal models. In addition, depending on the structure, curcumin might directly affect the target molecules^1,17,18^. Reportedly, curcumin affects diverse molecular targets and, possibly, binds protein kinases and other enzymes and molecules; however, the molecular mechanisms remain scarcely known. In contrast, quercetin binds protein kinases, p-glycoprotein and topoisomerases II^17–19^. Furthermore, curcumin (and the metabolites in the liver and kidneys) was detected in the liver, kidneys, heart and intestinal mucosa when high-dose curcumin (340 mg/kg) was orally administered to rats^20^. Although the curcumin concentrations of tissues were higher in plasma, the tissue distribution of curcumin in visceral WAT remains unknown.

Thus, this study aims to elucidate the preventive effects of dietary curcumin on lifestyle-related diseases in mice fed a diet reflective of a Western-style diet of humans, containing curcumin. As WAT is an essential target organ for preventing metabolic syndrome, we investigated the tissue distribution of curcumin as well as gene expression and lipidomic profiles in WAT in Western diet-induced obese mice.

## Results

### Curcumin accumulation in eWAT and reduction of lipid peroxidation markers’ levels in obese mice

In C57/BL6J mice, the Western diet, reflective of a Western-style diet of humans, increased the body weight, weight of the liver and visceral fat (the sum of epididymal, peri-renal, retroperitoneal and mesenteric fats) and hepatic lipid contents (Tables S1–S3; Fig. S1a). The dietary intake of curcumin was analogous to flavonoid quercetin, which suppressed the Western diet-induced obesity at 0.05% (*w*/*w*) of the diet; however, the bioavailability of curcumin remained low. Although the addition of 0.1% (*w*/*w*) curcumin did not considerably reduce the body weight gain and hepatic and liver fat accumulation, it substantially reduced the levels of non-fasting blood glucose and oxidative stress markers in the liver and eWAT (Fig. S1c; Table S3). In addition, curcumin did not affect the daily food intake of mice fed either diet (Fig. S1b). Thus, we assessed the tissue distribution of curcumin and the gene expression and lipid profiles in eWAT after the 16-week feeding of 0.1% curcumin in a Western diet on mice to elucidate the effect of curcumin on visceral WAT in diet-induced obese mice. Table 1 presents the body and tissue weights, levels of oxidative stress markers and blood constituents in each group in which we determined gene expression and lipid profiles of eWAT. Figure S2 shows the body weight, food consumption and blood glucose levels during the feeding period. Although curcumin did not considerably reduce the weights of visceral fat, including eWAT, it reduced malondialdehyde levels in eWAT of Western diet-induced obese mice (Table 1).

**Table 1:** Curcumin reduced the levels of blood glucose and lipid peroxidation markers in Western diet-induced obese mice.

	Control diet	Western diet	Western diet + 0.1% curcumin
Body weight (g)	41.51 ± 1.55^a^	47.49 ± 0.79^b^	44.93 ± 0.83^ab^
Liver weight (g)	1.62 ± 0.07^a^	3.44 ± 0.20^b^	3.08 ± 0.25^ab^
Kidney weight (g)	0.37 ± 0.02	0.38 ± 0.01	0.39 ± 0.01
Pancreas weight (g)	0.33 ± 0.03	0.33 ± 0.02	0.34 ± 0.02
(eWAT (g/mouse)) Visceral fat (g/mouse)	(2.09 ± 0.18) 3.71 ± 0.33	(2.42 ± 0.08) 4.48 ± 0.22	(2.45 ± 0.07) 4.17 ± 0.17
**Malondialdehyde in livers (nmol/mg protein)**	**1.96** ± **0.15**^**a**^	**3.42** ± **0.54**^**b**^	**0.74** ± **0.16**^**c**^
**Malondialdehyde in eWAT (nmol/mg protein)**	**4.38** ± **0.20**^**a**^	**9.02 **±** 0.83**^**b**^	**3.85** ± **0.42**^**a**^
**Blood glucose (mg/dL)**	**152** ± **7**^**a**^	**201** ± **6**^**b**^	**162** ± **8**^**a**^
Plasma insulin (ng/mL)	2.07 ± 0.49	4.02 ± 0.74	3.08 ± 0.42
Plasma cholesterol (mg/dL)	83.7 ± 10.3^a^	208.9 ± 9.8^b^	211.4 ± 14.9^b^
Plasma triglyceride (mg/dL)	66.2 ± 4.9^a^	55.2 ± 4.2^ab^	46.1 ± 1.2^b^
Plasma NEFA (mEq/dL)	0.68 ± 0.11^a^	0.54 ± 0.05^ab^	0.34 ± 0.03^b^
**Plasma 8-isoprostane (pg/mL)**	**166.1** ± **18.5**^**a**^	**341.3** ± **53.9**^**b**^	**245.5** ± **26.7**^**ab**^
Plasma TNF-α (pg/mL)	3.63 ± 0.44	12.76 ± 5.97	6.10 ± 1.14
Plasma IFN-γ (pg/mL)	4.60 ± 0.94	48.59 ± 26.25	18.89 ± 11.04
Plasma leptin (ng/mL)	3.69 ± 0.83^ab^	5.38 ± 0.46^a^	3.35 ± 0.32^b^

C57BL/6 J mice were fed the control AIN93G diet or a Western diet supplemented with either 0% or 0.1% curcumin for 18 weeks. Values are expressed as mean ± SEM (8–9 mice/group). Different superscripts (a, b, c) indicate significant differences (*P* < 0.05, two-sided).

**Figure 1:**
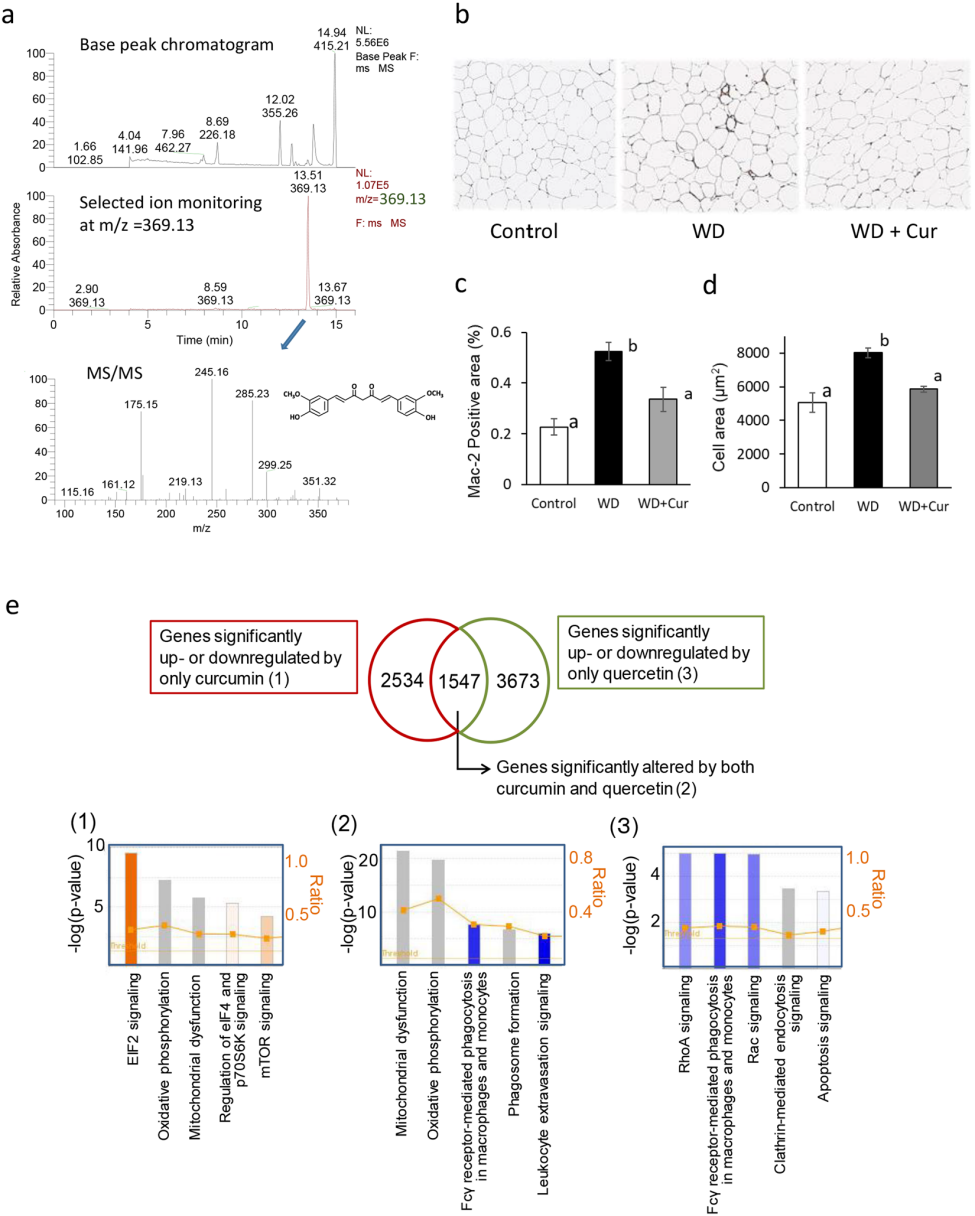
The effect of curcumin on macrophage accumulation and gene expression in epididymal white adipose tissue (eWAT) of Western diet-induced obese mice. C57BL/6J mice were fed AIN93G diet (Control), a Western diet (WD) or a Western diet supplemented with 0.1% curcumin (WD + Cur) for 16 weeks. (**a**) Representative base peak, selected reaction monitoring (369.13 *m*/*z*), and tandem mass spectrometry chromatogram of curcumin in eWAT of mice fed WD + Cur for 16 weeks. (**b**) Representative photomicrographs showing eWAT stained with the anti-Mac2 antibody (Mac2). (**c**) The proportion of the Mac2-stained area and (**d**) eWAT area evaluated by hematoxylin–eosin staining. Values are expressed as mean ± SEM of 7–9 mice in each group. Different superscripts indicate significant differences (*P* < 0.05, two-sided). (**e**) top five canonical pathways of genes considerably upregulated or downregulated by curcumin (1), both curcumin and quercetin (2) and quercetin (3) in eWAT of diet-induced obese mice. The most considerably altered functions in the dataset were determined by the ingenuity pathway analysis. Genes substantially upregulated or downregulated by quercetin in eWAT of obese mice were previously described^12^ (refer Table S4 as well).

In this study, we first analysed curcumin and its metabolites in eWAT. Selected ion monitoring and tandem mass spectrometry (MS/MS) confirmation determined curcumin aglycone in eWAT of mice fed a curcumin-supplemented Western diet (Fig. 1a). After 16 weeks of feeding, curcumin accumulated in eWAT (concentration, 299 ± 113 pmol/g; *n* = 6) in diet-induced obese mice. Consistent with the commercially available curcumin used in this study we detected smaller amounts of dimethoxy curcumin and bisdimethoxy curcumin in eWAT. Meanwhile, only trace amounts of curcumin glucuronide, a major metabolite, were determined in eWAT and plasma (data not shown).

### Suppression of macrophage accumulation and alteration of the gene expression profile in eWAT by curcumin

As macrophages were accumulated and classically activated in adipose tissue in obese mice, we evaluated the accumulation of macrophages in eWAT with anti-Mac2 staining (Fig. 1b). Figure 1c shows the Mac2-positive area in eWAT of mice fed AIN93G, Western diet and curcumin-supplemented Western diet, respectively (different superscripts signify substantial differences among the three groups (control, Western diet [WD], and curcumin-supplemented Western diet [WD + Cur])). Curcumin considerably suppressed the Mac2-positive macrophage accumulation in eWAT of diet-induced obese mice. In addition, curcumin decreased the area of each adipocyte in eWAT of Western diet-fed mice (Fig. 1d). The comprehensive gene expression analysis of a DNA microarray revealed the profile of dietary curcumin intake–induced gene expression alterations in eWAT of diet-induced obese mice. Then, we compared genes that were considerably upregulated or downregulated by curcumin with those altered by anti-oxidative flavonoid quercetin in eWAT of obese mice (Fig. 1e; Table S4). Among altered genes, 2534 genes were upregulated or downregulated only by curcumin (*n* = 5; see Methods section for statistical details). The ingenuity pathway analysis demonstrated that curcumin predominantly altered the gene expression associated with eukaryotic translation initiation factor 2 (eIF2) signalling, which is involved in the global protein synthesis and endoplasmic reticulum (ER) stress signalling (Fig. 1e(1) and Table S4(1)). In addition, the gene expression related to mitochondrial oxidative phosphorylation was considerably altered by curcumin (Fig. 1e(1) and (2); Tables S4(1) and (2)). Besides, the canonical pathways related to macrophages and leukocytes were predicted to be downregulated by both curcumin and quercetin (Fig. 1e(2); Table S4(2)). Quercetin altered the expression of more genes related to macrophages and integrin-mediated Rho and Rac signalling, implying that curcumin was less effective in suppressing the inflammation of eWAT in Western diet-induced obese mice (Fig. 1d(3); Table S4(3)).

Curcumin was predicted to suppress biological functions related to macrophages, neutrophils, T cells, natural killer cells and mast cells, as well as the synthesis and production of the reactive oxygen species (Table 2). Moreover, the cytochrome c oxidase assembly factor Surf, TNF-α, IFN-γ and molecules regulated by TNF-α and/or IFN-γ were predicted to be upstream regulators inhibited by curcumin (Table S5). Notably, peroxisome proliferator-activated receptor-γ (PPAR-γ), which plays a vital role in obesity and insulin resistance, was predicted to be activated by curcumin (Table S5).

**Table 2:** Predicted biological functions suppressed by curcumin in epididymal white adipose tissue of Western diet-induced obese mice (also refer Table S4).

Predicted biological function decreased by curcumin	*P*	Activation *z*-score
Quantity, activation, homing, uptake of **cells**	3.85E–03–4.85E–02	−3.274 to −2.133
Quantity, cell movement, homeostasis, activation, differentiation, homing, immune response, phagocytosis of **leukocytes**	2.59E–03–4.73E–02	−3.396 to −2.012
Homing of **lymphocytes**	4.63E–02	−2.671
Cell movement, activation, response, migration, engulfment of **myeloid cells**	8.11E–04–4.39E–02	−3.627 to −2.018
Engulfment, phagocytosis of **blood cells**	1.08E–02	−2.524 to −2.445
Phagocytosis of **red blood cells**	2.12E–02	−2
Cell movement, migration, response, degranulation, accumulation, Cell viability, engulfment, cell rolling, cytotoxicity of **phagocytes**	9.36E–04–4.20E–02	−3.695 to −2.028
Cell movement of **antigen-presenting cells**	1.03E–02	−2.244
Cell movement, chemotaxis, orientation of **macrophages**	3.90E–03–2.66E–02	−2.7 to −2.198
Cell movement, cell rolling of **granulocytes**	6.52E–03–3.39E–02	−3.856 to −2.54
Cell movement, homing, chemotaxis, recruitment, cell rolling of **neutrophils**	1.81E–03–4.97E–02	−4.286 to −2.044
Quantity o**f peripheral T lymphocyte**	2.34E–03	−2.236
Cell movement of **natural killer cells**	2.78E–02	−2.036
Recruitment of **mast precursor cells**	2.12E–02	−2
Degranulation, adhesion of **mast cells**	7.45E–03–1.01E–02	−2.425 to −2.079
**Inflammatory response**	1.11E–02	−2.483
Synthesis, production of **reactive oxygen species**	3.06E–05–1.29E–04	−3.136 to −2.62
Production of **superoxide**	4.19E–03	−2.319
Synthesis of **protein**	2.51E–02	−2.236

### Suppression of eIF2α phosphorylation and weakly suppressed inflammation by curcumin

**Figure 2:**
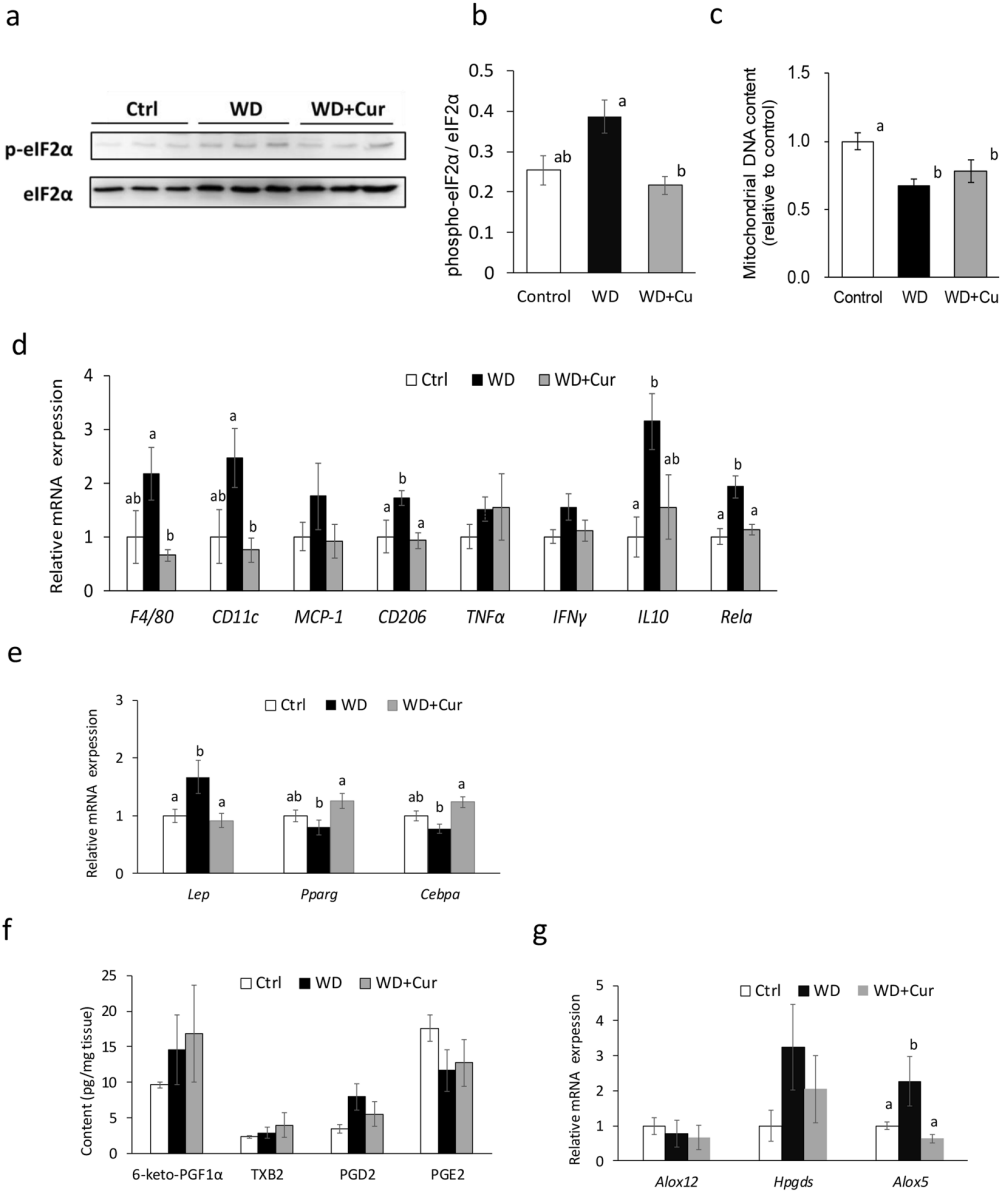
Curcumin suppresses eukaryotic translation initiation factor 2 (eIF2) phosphorylation and improves the expression of nuclear factor-κB (NF-κB) p65, leptin and peroxisome proliferator-activated receptor-γ (PPAR-γ) in epididymal white adipose tissue (eWAT) of Western diet-induced obese mice. Mice were fed AIN93G diet (Control or Ctrl), a Western diet (WD) or a Western diet supplemented with 0.1% curcumin (WD + Cur) for 16 weeks. (**a**) Immunoblot of eukaryotic translation initiation factor 2 (eIF2) and phospho-eIF2 (p-eIF2) in eWAT. (**b**) The ratio of eIF2 phosphorylation in eWAT. Both eIF2 and phospho-eIF2 were quantified using ImageQuant LAS500 (GE Healthcare; *n* = 5). (**c**) mitochondrial DNA content (*n* = 8–9) and (**d** and **e**) gene expressions determined by qRT-PCR (*n* = 9). (**f** and **g**) The eicosanoid content determined by LC-MS/MS (*n* = 3 [control] or *n* = 5) in eWAT. Values are expressed as mean ± SEM in each group. Different superscripts imply significant differences (*P* < 0.05, two-sided). Full-length Western blot representative images are presented in Fig. S3. 6-keto-PGF1α, 6-keto-prostaglandin F1α; TXB_2_, thromboxane B_2_.

The ER stress, which is associated with inflammation in adipose tissue in obese subjects, increased in WAT of obese subjects, suggesting the induction of lipolysis and increment of plasma lipid levels. The ER stress induces eIF2α phosphorylation and increases the expression of genes related to the ER stress responses. As curcumin probably affected eIF2 signalling, we assessed eIF2α phosphorylation in eWAT of mice fed a control diet, Western diet, or curcumin-supplemented Western diet. Curcumin substantially suppressed eIF2α phosphorylation in eWAT of obese mice (Fig. 2a and b; Fig. S3). In addition, A decline in the mitochondrial DNA content reduces β-oxidation and impairs mitochondrial oxidative phosphorylation. Although curcumin changed the expression of genes related to mitochondrial oxidative phosphorylation, a decline in the mitochondrial DNA copy number in eWAT of obese mice was not considerably improved by curcumin (Fig. 2c). The comprehensive gene expression analysis suggested that curcumin suppressed immune cell accumulation, as well as TNF-α and IFN-γ signalling, in eWAT of obese mice. In addition, the RT-PCR analysis revealed that curcumin suppressed the macrophage marker F4/80, dendritic cell and M1 macrophage marker CD11c and oxidative stress-sensitive transcription factor NF-κB p65 (encoded by *Rela*; Fig. 2d). Moreover, curcumin suppressed the expression of the M2 macrophage marker CD206 in eWAT of obese mice (Fig. 2d). However, it did not sufficiently suppress the expression of TNF-α and IFN-γ in eWAT of obese mice (Fig. 2d). The PPAR-γ expression, which was anticipated to be an activated upstream regulator by the comprehensive gene expression analysis (Table S5), was substantially increased by curcumin in eWAT of obese mice (Fig. 2e). Plasma leptin levels, which were considerably elevated in diet-induced obese mice, were reduced by curcumin feeding (Table 1). In addition, an increment in the leptin expression in eWAT was considerably decreased by curcumin (Fig. 2e). Although the levels of some eicosanoids, such as PGD_2_ and PGE_2_, have been reported to be related to inflammation in adipose tissue in obese subjects, eicosanoid levels in eWAT were not substantially altered by Western diet or curcumin consumption in this study (Fig. 2f). Apparently, Arachidonate 5-lipoxygenase (ALOX5) is involved in the synthesis of leukotriene B_4_ (LTB_4_) and lipoxin A_4_ (LXA_4_). Although LTB_4_ and LXA_4_ were undetectable in eWAT in all three groups, the *Alox5* expression was substantially elevated in eWAT of obese mice and decreased by curcumin (Fig. 2g; Table S7).

### Reduction of some lipid levels and related gene expressions by curcumin

**Figure 3:**
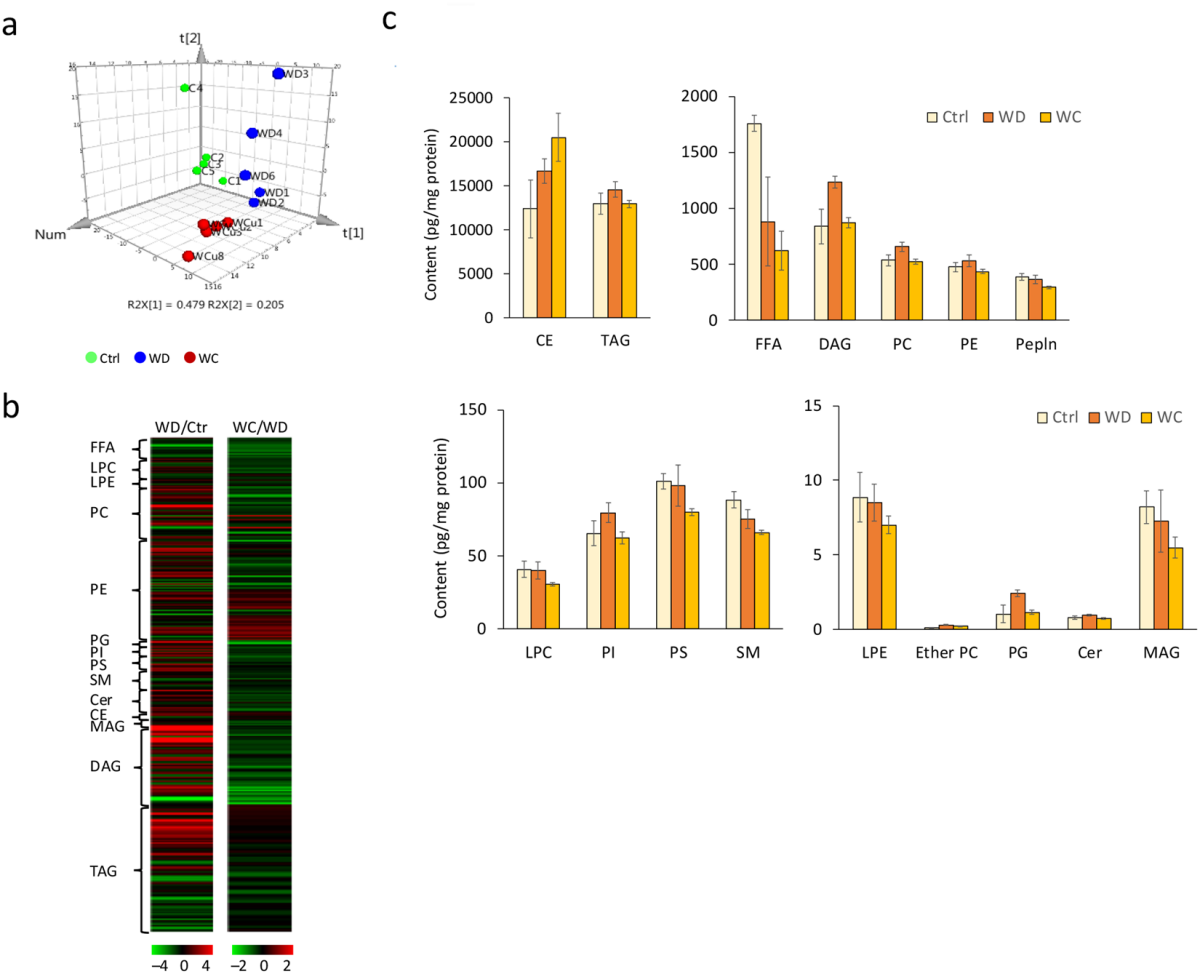
The lipidomic analysis of epididymal white adipose tissue (eWAT) of mice fed various diets. Mice were fed AIN93G diet (Control or Ctrl), or a Western diet (WD) or a Western diet supplemented with 0.1% curcumin (WD + Cur) for 16 weeks. The lipidomic analysis of eWAT was performed using supercritical fluid chromatography/tandem mass spectrometry. (**a**) The principal component analysis of the lipidome, (**b**) heat map of lipid species and (**c**) contents of lipid classes in eWAT. Values are expressed as mean ± SD for each group (n = 5). Colour scales for the heat map signify the levels of each lipid. Data were log_2_ transformed and the mean value of five biological replicates is presented. CE, cholesterol ester; TAG, triacylglycerol; FFA, free fatty acid; DAG, diacylglycerol; PC, phosphatidylcholine; PE, phosphatidylethanolamine; PEp, plasmenyl-phosphatidylethanolamine (phosphatidylethanolamine plasmalogen); LPC, lysophosphatidylcholine; PI, phosphatidylinositol; PS, phosphatidylserine; SM, sphingomyelin; LPE, lysophosphatidylethanolamine; PCe, plasmanyl-phosphatidylcholine; PCp, plasmenyl-phosphatidylcholine (PC plasmalogen); PG, phosphatidylglycerol; Cer, ceramide and MAG, monoacylglycerol. Also refer Table S6.

**Figure 4:**
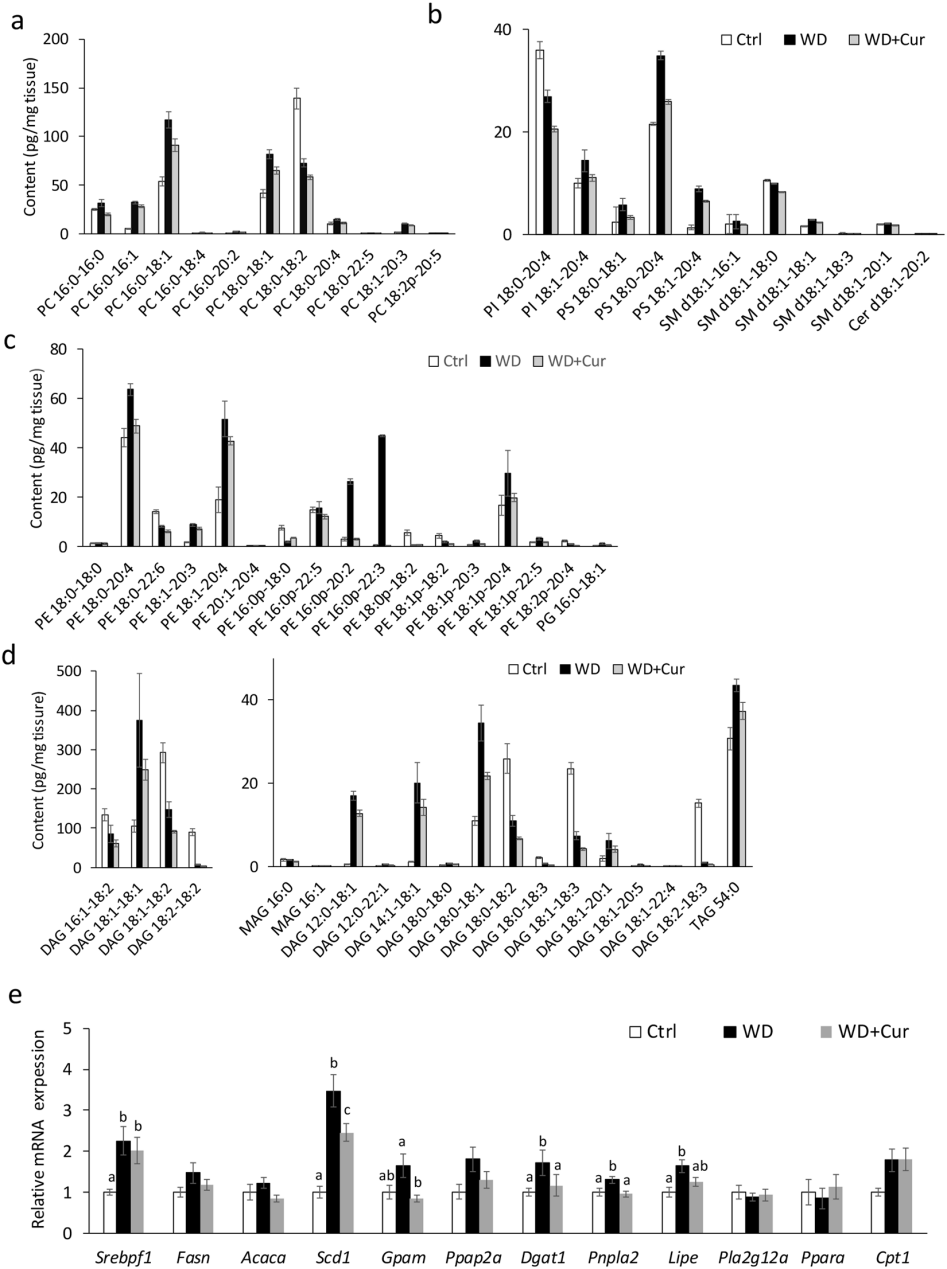
Curcumin alters the lipid content and related gene expression in epididymal white adipose tissue (eWAT) of obese mice. Mice were fed AIN93G diet (Control or Ctrl), a Western diet (WD) or a Western diet supplemented with 0.1% curcumin (WD + Cur) for 16 weeks. (**a**) Lipid contents (*n* = 5) were significantly different between the WD and WD + Cur groups (*P* < 0.05; *q* < 0.1; also refer Table S5). (**b**) The gene expression (*n* = 9) in eWAT. Values are expressed as mean ± SD (**a**–**d**) or SEM (**e**) for each group. Different superscripts imply significant differences (*P* < 0.05, two-sided).

**Figure 5:**
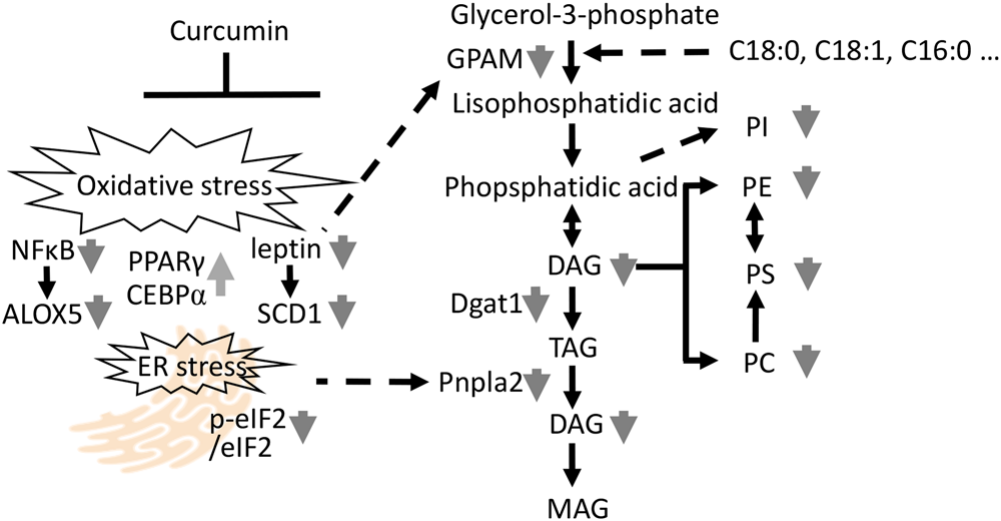
Suggested physiological effects of curcumin on adipose tissue in diet-induced obese mice.

Using supercritical fluid chromatography triple quadrupole mass spectrometry (SFC/MS/MS) in multiple reactions monitoring (MRM) mode, we performed the lipidomic analysis of eWAT. Figure 3a shows the results of the principal component analysis (PCA) of the lipidome in eWAT of mice fed a control diet, Western diet, or curcumin-supplemented Western diet. We used the PCA to assess the data structure obtained by SFC/MS/MS; the variance of samples differences was successfully captured in PCA score plots. In control mice and Western diet-induced obese mice, the composition of each lipid class in eWAT varied markedly, implying an impact of lipid compositions of diets (Fig. 3b). The control AIN93G diet comprised 7% purified soybean oil, which is rich in linoleic acid (18:2) and linolenic acid (18:3), whereas the Western diet comprised 20% butter, which is rich in oleic acid (18:1), myristic acid (14:0), palmitic acid (16:0) and stearic acid (18:0) and 1% purified soybean oil. Notably, the fatty acid composition of lipids in eWAT, such as triacylglycerol (TAG), diacylglycerol (DAG), phosphatidylcholine (PC), phosphatidylethanolamine (PE), lysophosphatidylcholine (LPC) and lysophosphatidylethanolamine (LPE) reflected that of the diet (Table S6). We observed significant differences (*P* < 0.05 and *q* < 0.05) in fatty acid content between control mice and Western diet-induced obese mice (Table S6; Fig. 4). Then, we compared the lipid content of eWAT between Western diet-fed mice and those fed a curcumin-supplemented Western diet to elucidate the effect of curcumin on the lipid profile of eWAT in obese mice. In addition, no significant differences were observed in lipid class contents between the groups (Fig. 3c). Figure 4a–d shows lipid molecules with differences in levels at *P* < 0.05 and *q* < 0.1. Curcumin reduced the content of some lipid classes, such as DAG, PC, PE and phosphatidylserine (PS; Fig. 4a,c and d), suggesting that curcumin suppressed the synthesis of DAGs and DAG-derived phospholipids in eWAT of diet-induced obese mice. Moreover, curcumin might have suppressed lipolysis of triglycerides in eWAT of obese mice. Next, we determined the expression of genes related to lipogenesis, lipolysis and lipid metabolism, implying that curcumin considerably suppressed the induction of mitochondrial glycerol-3-phosphate acyltransferase 1 (GPAT1, encoded by *Gpam*) and diacylglycerol *O*-acyltransferase 1 (DGAT1), related to triglyceride synthesis in eWAT of obese mice (Figs 4d and 5). Furthermore, curcumin substantially suppressed the expression of *Pnpla2*, which encodes adipose triglyceride lipase (ATGL; Figs 4e and 5). This study suggested that dietary curcumin suppressed the triglyceride synthesis and lipolysis and decreased the levels of certain DAGs and related phospholipids in visceral adipose tissue (Fig. 5).

## Discussion

Ejaz *et al*. reported that the supplementation with 0.05% curcumin considerably decreased the body weight gain in high-fat diet-fed mice^21^. In this study, 0.05% quercetin, but not 0.1% curcumin, considerably reduced the body weight gain and fat accumulation in visceral adipose tissue of Western diet-induced obese mice^13,14^. Although the suppressive effect of curcumin on obesity and metabolic syndrome was weaker than that of quercetin, dietary curcumin markedly suppressed the levels of lipid peroxidation markers in the plasma, liver and eWAT in obese mice. In addition, we determined that curcumin accumulated at a level of approximately 300 pmol/g in eWAT of mice after the chronic intake of a curcumin-supplemented Western diet. Perhaps, curcumin directly affected eWAT and decreased the oxidative stress increased by a Western diet in mice. Previously, we fed mice a Western diet supplemented with 0.05% quercetin as described for curcumin in this study; After 18 weeks of feeding, we detected quercetin in eWAT of obese mice at approximately 5 pmol/g, whereas glucuronidated and/or sulphated quercetin metabolites accumulated at approximately 190 pmol/g^14^. Reportedly, curcumin glucuronide is a primary metabolite of curcumin, although the oral bioavailability of curcumin is low^20,22^. In the plasma, only trace amounts of curcumin glucuronide, but not curcumin, were detected. Pan *et al*. reported that orally administered curcumin attained the maximum concentration at 1 h and declined to below the detection limit within 6 h in the plasma of mice^23^. Probably, curcumin is quickly degraded or excreted and, thus, eliminated from the plasma.

The comprehensive gene expression analysis illustrated the effect of curcumin on eWAT of obese mice. Quercetin is a natural anti-oxidative polyphenol that exhibits low specific manifold bioactivity. Both curcumin and quercetin exhibit potent antioxidant activity *in vivo*^14^. A comparison of the effects of curcumin and quercetin on gene expression profiles presented numerous differences between these antioxidants. Like quercetin, curcumin suppressed the gene expression related to the functions and signalling of immune cells such as macrophages, neutrophils, T cells, natural killer cells and mast cells in eWAT of obese mice. While quercetin substantially reduced the expression of immune cell markers in eWAT and plasma levels of inflammatory cytokines TNF-α and IFN-γ, curcumin only suppressed the number and expression of macrophages^14^. Despite an increment of the M1-to-M2 macrophage ratio in eWAT of mice fed a high-fat diet, the expression of anti-inflammatory M2 macrophages increased in the same tissue. In addition, curcumin suppressed the expression of both M1 and M2 macrophage markers, and its suppressive effect on immune cell accumulations and activations was weaker than that of quercetin in eWAT of obese mice. Conversely, quercetin, but not curcumin, augmented the PPAR-α expression, which promotes β-oxidation and lowers circulating lipid levels, in the liver. Thus, perhaps, it suppressed the lipid accumulation in the liver and visceral WAT of Western diet-induced obese mice^13^. Suppressing the lipid accumulation was, probably, more effective than alleviating the oxidative stress in inhibiting the immune cell accumulations and activations in eWAT of obese mice. Furthermore, curcumin substantially suppressed the expression of the oxidative stress-inducible transcription factor NF-κB p65 and NF-κB–regulated ALOX5 in eWAT of obese mice^24–26^.

The adipokine leptin expression increased in adipose tissue of obese subjects, stimulating the production of pro-inflammatory cytokines, such as TNF-α and IFN-γ, by macrophages, monocytes and T cells^27,28^. Perhaps, the antioxidant lipoic acid suppressed the leptin expression by reducing the DNA-binding activity of the transcription factor Sp1 in adipocytes^29^. A study demonstrated Sp1 to be oxidative stress-inducible transcription factor^30^. Curcumin might suppress the leptin expression by inhibiting the transcriptional activity of Sp1 through the anti-oxidative property of eWAT. Besides, TNF-α suppresses the expression and activity of PPAR-γ in adipocytes and stimulates insulin resistance; NF-κB, activated by TNF-α and oxidative stress, reduces the transcriptional activity of PPAR-γ^31,32^. Moreover, curcumin tends to induce the PPAR-γ expression by decreasing the oxidative stress and NF-κB activity. Of note, C/EBPα co-localises and cooperates with PPAR-γ in adipocytes^33^. Perhaps, the induction of PPAR-γ and C/EBPα by curcumin contributes to augment the insulin sensitivity in eWAT of obese mice. In this study, the *Alox5* expression was considerably elevated in eWAT of Western diet-fed mice and decreased by curcumin; however, LTB_4_ and LXA_4_ were undetectable in eWAT of all three groups. Assumedly, as the Western diet does not induce severe obesity and symptoms, it did not substantially elevate the levels of LTB_4_ and LXA_4_.

Conversely, curcumin changed the expression of genes related to eIF2 signalling. The obesity-induced ER stress stimulates eIF2α phosphorylation, causing global attenuation of the mRNA translation and selective increment in the translation of several RNAs related to ER stress responses^34,35^. Moreover, the ER stress induces chronic inflammation, insulin and leptin resistance and lipolysis in WAT^36,37^. In this study, curcumin reduced eIF2α phosphorylation in eWAT of Western diet-induced obese mice. Wang *et al*. reported that intra-gastrically administered curcumin suppressed the ER stress and lipolysis in adipose tissue, accounting for increment in circulating free fatty acid (FFA) levels and hepatic TG and DAG contents and prevented hepatic insulin resistance in a high-fat diet-fed early-stage insulin resistance mouse model^38^. When we treated differentiated 3T3-L1 cells for 1 h with curcumin alone followed by a combination with the ER stress–inducer thapsigargin for 1 h, 1 μM curcumin, but not 20 μM curcumin, marginally suppressed eIF2α phosphorylation (data not shown). In this study, curcumin did not adequately affect the plasma lipid levels; however, the reactive oxygen species reportedly enhance the ER stress in adipocytes^34^. Perhaps, curcumin suppresses the ER stress and improves the expression of genes, such as NF-κB, leptin and PPAR-γ, by alleviating the oxidative stress rather than directly suppressing eIF2α phosphorylation.

Apparently, excess lipid accumulation changes the lipid profile of WAT, and, reportedly, high-fat diet results in the dysregulation of lipolysis and lipid metabolism in eWAT and subcutaneous adipocytes^39^. Lipogenesis is improved in adipose tissue of obese mice, and lipogenic genes are upregulated in adipose tissue of obese people^40–42^. Pietilainen *et al*. performed the lipidomic analysis of human adipose tissue in twin pairs discordant for obesity and reported that the compositions of membrane phospholipids varied between ‘lean’ and ‘obese’ people; besides, they suggested that the lipid composition altered to adapt to the adipose tissue expansion related to the positive energy balance^43^. Anti-oxidative polyphenols might affect the lipid profile of WAT in diet-induced obese mice. In addition, SFC/MS/MS could facilitate high-throughput, high-resolution and simultaneous analysis of polar lipids; it is suitable for the lipidomic analysis of eWAT comprising various acylglycerols and phospholipids^44–46^. Our lipidomic analysis revealed that the dietary lipid composition affected the composition of acylglycerols and glycerolipids in eWAT of mice. The dietary intake of curcumin decreased the levels of some acylglycerols or glycerolipids in eWAT of Western diet-fed mice. Curcumin did not substantially inhibit the expression of fatty acid synthase and the transcriptional factor sterol regulatory element-binding protein 1c (SREBP1c), but it reduced the GPAT1 expression, a rate-limiting enzyme of TAG biosynthesis and initiator of glycerolipid synthesis and DGAT1 in eWAT of obese mice. Apparently, GPAT1 regulates the fatty acid composition of phospholipids^47^. Our results suggest that curcumin suppressed the synthesis of glycerolipids and the metabolites DAG and TAG from dietary fatty acids in eWAT. Gaidhu *et al*. reported that the high-fat diet intake augmented the ATGL expression, which initiated lipolysis by cleaving fatty acids from TAG and DAG content in visceral WAT in mice^39^. The suppression of the GPAT1 and ATGL expression, perhaps, contributes to the suppressive effect of curcumin on the levels of DAG and glycerolipid molecules in eWAT of Western diet-induced obese mice.

Stearoyl-CoA desaturase 1 (SCD1) catalyses the biosynthesis of monounsaturated fatty acids. Although the lipidomic analysis did not evidently reveal an increase in the ratio of unsaturated fatty acids, such as palmitoyl and stearoyl acids, curcumin considerably suppressed the *Scd1* expression in eWAT of obese mice, which might contribute to decreasing palmitoleic and oleic acid contents in DAG and glycerolipid molecules. SCD1 comprises a leptin response element containing both SP1 and AP1 consensus binding sites in the promoter region, and its transcription was suppressed by leptin^48^. Perhaps, curcumin suppressed the SCD1 expression by inhibiting the leptin expression in eWAT of obese mice. Reportedly, GPAT1 is regulated at both the transcriptional and posttranscriptional levels^49^, and the *Gpat1* expression is decreased in adipose tissue of leptin-deficient mice^40^. DGAT1 catalyses the TAG synthesis from DAG in the ER, and the DGAT deficiency reduced triglyceride levels and leptin expression in WAT and enhanced the leptin sensitivity in mice^50^. Although SREBP-1 and PPAR-γ are major transcriptional regulators of GPAT1 and DGAT1, respectively, curcumin did not inhibit their expression^47,51^. The GPAT1 and DGAT1 expression might be suppressed by a reduction in the leptin expression or other mechanisms. In addition, AMP-activated protein kinase downregulates GPAT1 and upregulates carnitine palmitoyltransferase-1 (CPT1), but curcumin did not affect the CPT1 expression. Reportedly, ATGL-knockout mice were protected against the fatty acid–derived ER stress in the liver^52^, and curcumin, probably, marginally suppressed eIF2 phosphorylation induced by the ER stress and reduced the levels of DAGs and DAG-derived glycerophospholipids by suppressing GPAT1 and ATGL expressions in eWAT of obese mice.In conclusion, curcumin marginally accumulated in eWAT after the chronic dietary intake; however, its metabolites were scarcely detected in the plasma and eWAT of Western diet-induced obese mice. Although the habitual intake of dietary curcumin did not considerably affect the visceral fat weight and plasma lipid levels, the global gene expression and lipid analysis demonstrated weak and manifold bioactive properties of curcumin in eWAT of obese mice. Curcumin changed the eIF2 signalling–related gene expression, suppressed ER-induced eIF2 phosphorylation and macrophage accumulations and enhanced the expression of NF-κB p65, leptin and other molecules through its anti-oxidative properties probably. Besides, curcumin reduced the levels of DAGs and DAG-derived glycerophospholipids, perhaps, by suppressing the GPAT1 and ATGL expressions that are associated with lipogenesis and lipolysis, respectively. Most manifold bioactivites were probably attributed to anti-oxidative properties of curcumin; however, curcumin could directly affect molecules and regulate some gene expressions in eWAT. Hence, these effects presumably contribute to the prevention of metabolic syndrome through dietary modification. The effects of higher doses of curcumin on diet-induced obese mice will be investigated in the next study; we will therefore confirm the effects of curcumin on immunometabolic alterations and adipocytokine and gene expression profiling in the eWAT and will also investigate glucose homeostasis in obese mice.

## Methods

### Animal experiments

We purchased C57BL/6 J mice (5-week-old, male) from the Charles River Japan Inc. (Ibaraki, Japan) which were maintained at 24 °C ± 1 °C and 55% ± 5% humidity under 12:12 h light–dark photocycles with lights on at 08:00 and provided *ad libitum* access to water and standard non-purified diet (NMF; Oriental Yeast Co., Tokyo, Japan). Mice were treated per the basic guidelines of the Ministry of Agriculture, Forestry, and Fisheries for laboratory animal studies, and all animal experiments were approved by the Institutional Animal Care and Use Committee of Food Research Institute, National Agriculture and Food Research Organization (permission numbers H26–007 and H27-002).

After 1-week acclimation, we divided mice into three groups (9 mice/group, 3/cage; body weights: 21.2 ± 0.4 g, 21.6 ± 0.2 g and 21.8 ± 0.4 g). One group was fed AIN93G (control group; Oriental Yeast Co.) and two were fed a Western-style modified AIN93G diet for 2 weeks. Tables S1 and S2 present the ingredient composition and energy term energy source of diets. After 2 weeks, the body weight of the control group was 24.3 ± 0.4 g, and other two groups were 26.0 ± 0.4 g and 26.0 ± 0.2 g. Then, we started feeding a Western diet supplemented with 0.1% *w*/*w* curcumin (Wako Pure Chemicals, Tokyo, Japan). We monitored the body weight and mean food consumption at 1-week and 2- or 3-day intervals, respectively (Fig. S2a and b). After 10 and 14 weeks of initiating curcumin feeding, we determined blood glucose levels (Fig. S2c). Next, all animals were sacrificed under anaesthesia, and the blood, liver, kidneys, adipose tissues and pancreas of each animal were collected immediately. Notably, the experimental conditions were determined per the preceding experiment (Fig. S1a–c).

### Measurement of Plasma and Tissue Levels of Curcumin and Metabolites

eWAT was homogenised with 50 mM phosphate buffer (pH 5.3). We extracted curcumin with acetonitrile, followed by quantification using an HPLC system (Shimazu) with an RF-10AXL Fluorescence Detector and excitation and emission wavelengths of 426 and 439 nm, respectively. The internal standard (quercetin) was measured using a UV–Vis detector (SPD-10AV VP) at 370 nm. Samples were applied to a Zorbax Bonus-RP -C18 column (4.6 × 150 mm, 5 μM; Agilent Technologies) and eluted with 20–100% acetonitrile in 0.2% formic acid for >30 min at a flow rate of 1 mL/min at 40 °C. We determined curcumin and its metabolites using a UHPLC-ESI-Q-Orbitrap-MS system (Ultimate3000 RSLC/LTQ Orbitrap Velos; Thermo Fisher Scientific). We used Xcalibur 2.2 software (Thermo Fisher Scientific) to conduct data acquisition and mass spectrometric evaluation. Samples were applied to a ACQUITY UPLC HSS C18 column (2.1 × 100 mm, 100 Å, 1.8 μM; Waters Co., Milford, MA) and eluted with 20–97% acetonitrile in 0.1% formic acid for >30 min at a flow rate of 0.3 mL/min at 40 °C. The electrospray ionisation (ESI) parameters were as follows: spray voltage, 3.0 and 3.5 kV for positive and negative modes, respectively; capillary temperature, 230 °C; sheet gas flow rate, 40 arbitrary units (AU) and auxiliary gas flow rate, 5 AU. In addition, we acquired full-scan mass spectra from 100 to 1000 *m*/*z* in the profile mode using resolutions of 100,000 and 60,000 for positive and negative modes, respectively. The selected ion monitoring was performed at 369.13 for curcumin and 543.15 for curcumin glucuronide, and the MC/MC activation type was collision-induced collisional dissociation. Furthermore, the normalised collision energy was set at 35% and the activation time was set at 10 ms.

### qRT-PCR Analysis and Western Blotting

We used TRIzol reagent (Thermo Fisher Scientific) and an RNeasy Midi Kit (Qiagen, Hilden, Germany) to extract the total RNA from eWAT. We performed qRT-PCR using an ABI PRISM 7000 Sequence Detection System or QuantStudio 3 System (Thermo Fisher Scientific) with Power SYBR Green Master Mix or PowerUp SYBR Green Master Mix (Thermo Fisher Scientific). Table S8 presents the primer sequences used for qRT-PCR. Notably, the relative amount of gene expression was normalised to that of *Gapdh* in the same cDNA, and mtDNA was amplified with primers for the mitochondrial cytochrome *b* gene and normalised to genomic DNA by amplifying the large ribosomal protein p0 (36B4) nuclear gene. Table S5 shows the primers. Data are expressed as mitochondrial genomes per diploid nuclei. Besides, denatured protein samples for Western blotting were separated by 12% SDS–polyacrylamide gel electrophoresis and electrophoretically transferred to a polyvinylidene difluoride membrane (Immobilon PVDF; Millipore). We detected proteins using specific antibodies (Cell Signaling Technology, Danvers, MA; #9721, RRID:AB_330951 and #9722, RRID:AB_2230924) and an Immobilon Western Chemiluminescent HRP Substrate (Millipore) and quantified using ImageQuant LAS500 (GE Healthcare).

### cDNA microarray analysis

We used GeneChip 3′ IVT Express Kit (Affymetrix) for the synthesis of fragmented biotin-labelled aRNA from the total RNA of each mouse’s eWAT. Then, we hybridised the aRNA to a GeneChip Mouse Genome 430 2.0 array (Affymetrix [Thermo Fisher Scientific]) and stained the array with a GeneChip Fluidics Station 450 (Affymetrix), and scanned (GeneChip Scanner 3000; Affymetrix) using GeneChip Operation Software Ver. 1.4 (Affymetrix). Notably, the data are deposited in the NCBI’s Gene Expression Omnibus^53^ and could be accessed through the GEO Series accession number GSE100388. Next, Microarray Suite 5.9 (MAS5; Affymetrix) and Subio platform version 1.19 (Subio, Kagoshima, Japan) were used for data analysis (n = 5 in each group). In addition, we used Welch’s one-way ANOVA and Welch’s *t*-test for statistical analyses of differentially expressed genes among the three groups (control, WD, and WD + Cur) or between two groups (Control and WD, WD and WD + Cur), respectively. We considered corrected *P* < 0.05 as statistically significant. The data for quercetin (GEO Series accession number GSE71367) were analysed similarly, and differentially expressed genes between the WD and WD + Cur groups were compared with those between the WD and WD + quercetin groups. Besides, the ingenuity pathway analysis (Ingenuity Systems, www.ingenuity.com) was used to determine the canonical pathways and biological functions that were most substantially affected in the extracted dataset. A *P* value denoting the probability that each canonical pathway and biological function for that dataset was because of a change in the given parameter alone were evaluated using a right-tailed Fisher’s exact test. Furthermore, the activation of canonical pathways, biological function and the functional or translational activation of upstream regulators were evaluated by an activation *z*-score; an absolute *z*-score of less (inhibited) or more than 2 (activated) was considered significant.

### Lipidomic analysis using SFC/MS/MS

We performed lipid extraction from eWAT (~20 mg) using the Bligh and Dyer’s method^54^ with minor modifications. Briefly, each frozen eWAT tissue was mixed with 1 mL of a solvent mixture (MeOH:CHCl_3_:H_2_O = 10:5:3 *v*/*v*/*v*) containing FFA 17:0 (5 μM), LPC 17:0 (0.25 μM), LPE 17:1 (50 μM), PC 17:0–17:0 (5 μM), PE 17:0–17:0 (4 μM), phosphatidylglycerol (PG) 17:0–17:0 (10 μM), PS 17:0–17:0 (10 μM), sphingomyelin (SM) d18:1–17:0 (0.05 μM), ceramide (Cer) d18:1–17:0 (0.05 μM), cholesterol ester (CE) 17:0 (50 μM), monoacylglycerol (MAG) 17:0 (5 μM), DAG 12:0–12:0 (0.5 μM) and TAG 17:0–17:0–17:0 (0.5 μM) as internal standards. We vortexed these tubes for 60 s, subjected to ultrasonic waves for extraction and centrifuged (16,000 × *g*, 4 °C, 5 min) to remove impurities and foreign substances. Then, we transferred 700 μL of the supernatant to another 2 mL Eppendorf tube, followed by adding 195 μL each of H_2_O and CHCl_3_ and vortexing this mixture for 60 s and centrifuging (16,000 × *g*, 4 °C, 3 min); 300 μL of the lower phase (chloroform phase) was placed in another 2-mL Eppendorf tube. Finally, 300 μL of MeOH was added to a tube, which was stored at −80 °C until analysis. The levels of each lipid molecular species were quantified using SFC/MS/MS in MRM mode; the SFC/MS/MS system comprised a SFC (ACQUITY UPC2; Waters Co.) and a triple quadrupole mass spectrometer (Xevo TQ-S micro; Waters Co.). The procedures for the quantitative lipidomics analysis are detailed elsewhere. Table S6 lists the lipidomics data.

### Statistical Analysis

We used Excel 2016 (Microsoft) and R-3.2.5 (R Foundation for Statistical Computing) for statistical analysis. Furthermore, SIMCA-P 13.0.3 (Umetrix, Sweden) was used for the PCA analysis with lipidomics data (n = 5 in each group). For lipidomic analysis, data are expressed as arithmetic mean ± SD. *P* values between two groups (control and WD, WD and WD + Cur) were determined using the Student’s *t*-test and showed in Table S6. False discovery rate–adjusted *q* values were evaluated using R package ‘qvalue’^55^ and Fig. 4a lists data significant at *P* < 0.05 and *q* < 0.1 between WD and WD + Cur.

Other data are expressed as arithmetic mean ± SEM. The significance of differences between three groups was determined using the ANOVA followed by two-tailed multiple *t*-tests with Holm’s correction. different superscripts signify substantial differences among the three groups (control, WD and WD + Cur). We considered *P* < 0.05 as statistically significant.

## Electronic supplementary material

Supplementary Information

Table S4

Table S6
