# Supplementary material for: Dietary Intake of Curcumin Improves eIF2 Signaling and Reduces Lipid Levels in the White Adipose Tissue of Obese Mice
Source: Sci Rep. 2018 Jun 13;8:9081. doi: 10.1038/s41598-018-27105-w (PMC5998036; doi:10.1038/s41598-018-27105-w)
Supplement: Supplementary file 1 — Supplementary Information [file 41598_2018_27105_MOESM1_ESM.docx]

**Supplementary Information**

**Dietary Intake of Curcumin Improves eIF2 Signaling and Reduces Lipid Levels in the White Adipose Tissue of Obese Mice**

**Masuko Kobori^1,*^, Yumiko Takahashi^1^, Hiroaki Takeda^2^, Masatomo Takahashi^2^, Yoshihiro Izumi^2^, Yukari Akimoto^1^, Mutsumi Sakurai^1^, Hideaki Oike^1^, Toshiyuki Nakagawa^3^, Masanori Itoh^3^, Takeshi Bamba^2^, Toshiyuki Kimura^1^**

^1^ Food Research Institute, National Agriculture and Food Research Organization, Tsukuba, Ibaraki 305-8642, Japan

^2^ Medical Institute of Bioregulation, Kyushu University, Fukuoka, Fukuoka 812-8582, Japan

^3^ Department of Neurobiology, Gifu University Graduate School of Medicine, Gifu, Gifu 501-1194, Japan

^*^ Corresponding author: [kobori@affrc.go.jp](mailto:kobori@affrc.go.jp)

**Table S1.** Ingredient composition (percentage by weight) of a AIN93G and a Western diet.

|  | **AIN93G** | **Western diet** |
| --- | --- | --- |
| Casein | 20% | 19.82% |
| L-cystine | 0.3% | 0.3% |
| Cornstarch | 39.7486% | 3.7458% |
| Pregelatinized cornstarch | 13.2% | 1.25% |
| Sucrose | 10.0% | 34.0% |
| Soybean oil | 7.0% | 1.0% |
| Cellulose powder | 5.0% | 5.0% |
| AIN-93G-mineral mix | 3.5% | 3.5% |
| AIN-93 vitamin mix | 1.0% | 1.0% |
| Choline bitartrate | 0.25% | 0.25% |
| Tetrabutylhydroquinone | 0.0014% | 0.0042% |
| Unsalted butter | 0% | 20.0% |
| Maltodextrin | 0% | 9.98% |
| Cholesterol | 0% | 0.15% |

**Table S2.** (percentage by energy) of a AIN93G and a Western diet.

|  | **AIN93G**  **(377kcal/100g)** | **Western diet**  **(450.8kcal/100g)** |
| --- | --- | --- |
| Protein | 19.0% | 15.8% |
| Fat | 16.7% | 39.9% |
| Carbohydrate | 64.3% | 44.3% |

a

b

c

**Figure S1.** Effect of a Western diet and a Western diet supplemented with 0.1% curcumin on body weight (a), food consumption (b) and blood glucose levels (c) in C57/BL6J male mice

5 week old male mice were divided into 3 groups and fed a AIN93G (control), a Western diet (WD), and a Western diet supplemented with 0.1% curcumin (WD+Cur) for 18 weeks.

**Table S3**

Effect of 18 week-feeding of a AIN93G, a Western diet, and a Western diet supplemented with 0.1% curcumin on C57/BL6J male mice

|  | Control diet | Western diet | Western diet+ 0.1% curcumin |
| --- | --- | --- | --- |
| Body weight (g) | 33.98 ± 1.11 ^a^ | 40.37 ± 2.19 ^b^ | 37.29 ± 1.81 ^ab^ |
| Liver weight (g) | 1.48 ± 0.04^a^ | 2.43 ± 0.31^b^ | 2.13 ± 0.23^ab^ |
| Kidney weight (g) | 0.38 ± 0.01 | 0.39 ± 0.01 | 0.35 ± 0.03 |
| Pancreas weight (g) | 0.26 ± 0.02 | 0.31 ± 0.02 | 0.27 ± 0.01 |
| Visceral fat (g/mouse) | 1.32 ± 0.38^a^ | 3.10 ± 0.46^b^ | 2.29 ± 0.51^ab^ |
| **Malondialdehyde in livers**  **(nmol/mg protein)** | **1.96 ± 0.15^a^** | **3.42 ± 0.54^b^** | **0.74 ± 0.16^c^** |
| Hepatic cholesterol (mg/g) | 2.17 ± 0.08^a^ | 7.16 ± 0.75^b^ | 4.10 ± 0.29^c^ |
| Hepatic triglyceride (mg/g) | 13.1 ± 1.5^a^ | 39.7 ± 2.1^b^ | 28.2 ± 4.2^b^ |
| Hepatic NEFA (mEq/g) | 1.45± 0.20^a^ | 4.60 ± 0.32^b^ | 3.16 ± 0.42^b^ |
| **Malondialdehyde in eWAT**  **(nmol/mg protein)** | **4.87 ± 0.36^a^** | **7.97 ± 0.84^b^** | **2.76 ± 0.23^c^** |
| **Blood glucose (mg/dL)** | **132 ± 8^a^** | **206 ± 18^b^** | **158 ± 10^a^** |
| Plasma insulin (ng/mL) | 0.69 ± 0.14 | 3.35 ± 0.65 | 2.57 ± 0.84 |
| Plasma cholesterol (mg/dL) | 84.2 ± 6.0^a^ | 160.0 ± 14.0^b^ | 129.4 ± 12.5^b^ |
| Plasma triglyceride (mg/dL) | 40.0 ± 5.4^a^ | 21.3 ± 1.6^b^ | 21.2 ± 2.6^b^ |
| Plasma NEFA (mEq/dL) | 0.54 ± 0.12 | 0.40 ± 0.01 | 0.48 ± 0.06 |
| **Plasma 8-isoprostane (pg/mL)** | **111.23 ± 9.91^a^** | **137.58 ± 21.98^a^** | **77.25 ± 5.23^b^** |
| Plasma TNFα (pg/mL) | 3.21 ± 0.27^a^ | 6.32 ± 0.49^b^ | 6.32 ± 0.44^b^ |
| Plasma leptin (ng/mL) | 0.41 ± 0.26^a^ | 2.12 ± 0.68^b^ | 0.95 ± 0.32^ab^ |

C57BL/6J mice were fed the control AIN93G diet, a Western diet, or a Western diet containing 0.1% curcumin for 18 weeks. Values are expressed as the mean±SEM of 7-9 mice in each group. Different superscripts indicate significant differences (P < 0.05, two-sided)

a

b

c

**Figure S2.** Effect of curcumin on the Western diet-induced body weight gain (a), food consumption (b), and the level s of blood glucose (c) in mice

6 week-old male mice were fed a AIN93 (1 group of 9 mice) or a Western diet (2 groups) for 2 weeks. Then one group of Western diet was changed to a Western diet containing 0.1% curcumin.

**Table S5.** Upstream regulators predicted to be activated or inhibited by curcumin in the eWAT of diet-induced obese mice, Related to Table 2.

| **Predictions** | |
| --- | --- |
| **Activated upstream regulator** | **Inhibited upstream regulator** |
| Pparg (34 target nolecules in dataset),  Lipe (10), Foxo1 (24) Esrra (21) | Surf1 (7 target molecules in dataset), Spi1 (12), mir-223 (24), Cd44 (6), Tnf (36), Klf3 (77) Map3k8 (24), Bid (4), Ifng (91), Ptges (4) |

*p* values < 0.05 were considered to be a significant dataset of the targets of each upstream regulator (Fisher's exact test). An absolute *z*‐score below (inhibited) or above (activated) 2 was considered significant.

LEPR, leptin receptor; NPC1, Niemann-Pick disease, type C; mir-223, microRNA‐223; ABCB6, ATP‐binding cassette, sub‐family B (MDR/TAP), member 6; ALOX5, arachidonate 5‐lipoxygenase; SPI1, transcription factor PU.1; SPIB, Spi‐B transcription factor (Spi‐1/PU.1 related).

**Figure S3.** Full-length Western blot representative image of eukaryotic translation initiation factor 2 (eIF2) and phospho-eIF2 shown in Figure 2(a).

the samples were derived from the same experiment and that gels/blots were processed in parallel.

**Table S7** Eicosanoids analyzed by LS-MS/MS.

|  | Compound | Precursor ion (*m/z*) | Product ion (*m/z*) |
| --- | --- | --- | --- |
| 1 | 2,3-dinor-8-iso-PGF2α | 325 | 237 |
| 2 | 6-keto-PGF1α | 369 | 163 |
| 3 | 20-COOH-LTB4 | 365 | 129 |
| 4 | 6-keto-PGE1 | 367 | 143 |
| 5 | 20-OH-LTB4 | 351 | 195 |
| 6 | TXB2 | 369 | 169 |
| 7 | PGF2α | 353 | 309 |
| 8 | PGE2 | 351 | 271 |
| 9 | 11-dehydro-TXB2 | 367 | 305 |
| 10 | 15-keto-PGF2α | 351 | 315 |
| 11 | LXB4 | 351 | 221 |
| 12 | PGD2 | 351 | 271 |
| 13 | LXA4 | 351 | 115 |
| 14 | LTD4 | 495 | 143 |
| 15 | LTC4 | 624 | 143 |
| 16 | LTF4 | 567 | 171 |
| 17 | LTE4 | 438 | 115 |
| 18 | PGA2 | 333 | 271 |
| 19 | PGJ2 | 333 | 271 |
| 20 | δ-12-PGJ2 | 333 | 271 |
| 21 | PGB2 | 333 | 175 |
| 22 | LTB4 | 335 | 195 |
| 23 | 12-keto-LTB4 | 333 | 179 |
| 24 | 15-deoxy-δ-12,14-PGJ2 | 315 | 271 |
| 25 | PGE2-d_4_ | 355 | 275 |

**Table S8** Primer sequences used for quantitative RT-PCR.

|  | Gene | Forward | Reverse |
| --- | --- | --- | --- |
| 1 | F4/80 (*Adgrel*) | ggaggacttctccaagcctatt | aggcctctcagacttctgctt |
| 2 | Cd11c (*Itgax*) | gagccagaacttcccaactg | tcaggaacacgatgtcttgg |
| 3 | MCP-1(*Ccl2*) | catccacgtgttggctca | gatcatcttgctggtgaatgagt |
| 4 | *Cd206* (*Mrc1*) | ccacagcattgaggagtttg | acagctcatcatttggctca |
| 5 | *Il10* | cagagccacatgctcctaga | tgtccagctggtcctttgtt |
| 6 | *Tnfa* | ccctcacactcagatcatcttct | gctacgacgtgggctacag |
| 7 | *Ifng* | actaccttcttcagcaacagcaa | ctggtggaccactcggatga |
| 8 | *Lep* | caggatcaatgacatttcacaca | gctggtgaggacctgttgat |
| 9 | PPARg (*Pparg*) | tcccgctgaccaaagcaaaggc | ccacggagcgaaactgacaccc |
| 10 | *Cebpa* | caaagccaagaagtcggtggacaa | tcattgtgactggtcaactccagc |
| 11 | *Rela* | tgcccagaccgcagtatc | ggattcgctggctaatgg |
| 12 | *Alox12* | ggggatggagaagctacagg | tccgcttcaaacagagtgc |
| 13 | *Hpgds* | cacgctggatgacttcatgt | aattcattgaacatccgctctt |
| 14 | *Alox5* | ttggtatcgccatgtaccg | gggctaccagcagcttga |
| 15 | *Srebpf1* | atccggcgcggaagctgtcggggtagcgtc | actgtcttggttgttgatgagctggagcat |
| 16 | *Fasn* | gctgctgttggaagtcagc | agtgttcgttcctcggagtg |
| 17 | *Acaca* | tgacagactgatcgcagagaaag | tggagagccccacacaca |
| 18 | *Scd1* | ttccctcctgcaagctctac | cagagcgctggtcatgtagt |
| 19 | *Gpam* | ggcatctcgtatgatcgcat | gcaaaatccactcggacgta |
| 20 | *Ppap2a* | ggccctcgatgtgatttg | aatggatatactttgcccaatttt |
| 21 | *Dgat1* | tcgtggtatcctgaattggtg | aggttctctaaaaataaccttgcatt |
| 22 | *Pnpla2* | ctgcctgccagactcaatg | ggtggtcatcaggtcctttg |
| 23 | *Lipe* | gcgctggaggagtgttttt | cgctctccagttgaaccaag |
| 24 | *Pla2g12a* | gactgtgacgaggagttccag | tcagctccctcctctatcacc |
| 25 | *Ppara* | ctgagaccctcggggaac | aaacgtcagttcacagggaag |
| 26 | *Cpt1* | accctgaggcatctattgacag | atgacatactcccacagatggc |
| 27 | *Gapdh* | atcccagagctgaacg | gaagtcgcaggagaca |
| 28 | *Gapdh2* | aagagggatgctgcccttac | ccattttgtctacgggacga |
| 29 | *36B4* | aggatatgggattcggtctcttc | tcatcctgcttaagtgaacaaact |
| 30 | *CytB* | tgagggggcttctcagtaga | ctgtttcgtggaggaagagg |

**Supplemental Experimental Procedures**

**Measurement of Blood Constituents and Lipid Peroxidation Markers.** Blood glucose levels were measured using a glucose test meter (Arkray Inc., Kyoto, Japan). Plasma total cholesterol, triglyceride, and NEFA were enzymatically measured using commercial kits (Wako Pure Chemicals Industries, Osaka, Japan). Plasma insulin, TNF-α, leptin, and IFN-γ concentrations were determined using commercial ELISA kits (Shibayagi, Gunma, Japan [for insulin] and eBioscience, San Diego, CA) according to the manufacturers’ instructions. The lipid peroxidation markers 8-isoprostane and malondialdehyde were measured using commercial kits (Cayman Chemical Company).

**Measurement of eicosanoids.** Standard eicosanoids and PGE_2_-d_4_ (internal standard) were mixed and diluted with ethanol:ultra pure water (1:1, v/v) as the standard solution. eWAT samples were frozen with liquid nitrogen and fractured using a multi-bead shocker (Yasui Kikai) three times at 1500 rpm for 10 s each. Five hundred microliters of saline, 500 μl of ethyl acetate, and 0.5 ng of PGE_2_-d_4_ were added to the samples, and the eicosanoids were then extracted using a multi-bead shocker. After centrifugation at 9100 × g for 5 min, the upper layer was recovered, and the ethyl acetate extraction was repeated twice. The organic solvent was removed using a centrifugal evaporator, and 250 μl of *n*-hexane and 500 μl of acetonitrile were added to the ethyl acetate fraction. Subsequently, the mixture was shaken for 5 min at 2500 rpm, and the upper layer was removed. The operation was repeated three times. The lower layer was evaporated and dissolved in 50 μl of methanol and diluted with 50 μl of ultra pure water as the samples for LC-tandem mass spectrometry (MS/MS) analysis. Eicosanoids in the sample were measured using an ACQUITY UPLC I-Class HPLC system (Waters) combined with a Xevo TQ-S triple quadrupole mass spectrometer (Waters). HPLC was conducted at 40°C using an *L-column2 ODS* Metal-free (2.0 × 150 mm, pore size 2 μm, CERI). Mobile phases (A) and (B) were 5 mmol/l ammonium formate:formic acid (1000:1, v/v) and acetonitrile, respectively. The chromatographic separation was achieved via a gradient elution as follows: %B = 10%, 10%, 25%, and 80% at 0, 1, 5, and 30 min, respectively. The flow rate was set at 0.4 ml/min. Samples were kept at 10°C, and 10 μl were injected. Tandem mass spectrometry analyses were conducted in the ESI negative ion mode, and the fatty acid metabolites were detected and quantified by multiple reaction monitoring (MRM). The conditions used to detect each compound by MRM are listed in Table S8. The source temperature was set at 500°C. The peaks were selected, and their areas were calculated using MassLynx ver. 4.1 (Waters). A signal-to-noise ratio of three was used as the limit of detection. Among the detected eicosanoids (6-keto-PGF_1α_, thromboxane B_2_ (TXB_2_), PGF_2α_, PGE_2_, PGD_2_, PGA_2_, and 15-deoxy-δ-12,14-PGJ_2_), four eicosanoids were quantified as shown in Figure 2f.
